# Burden of alcohol use disorder in Balkan countries

**DOI:** 10.1093/eurpub/ckac130.190

**Published:** 2022-10-25

**Authors:** J Todorovic, M Santric- Milicevic, Z Terzic-Supic, A Stevanovic, Z Stamenkovic, N Terzic, L Scepanovic, T Albreht, S Stoisavljevic

**Affiliations:** 1 Institute of Social Medicine, Medical Faculty, University of Belgrade, Belgrade, Serbia; 2 Institute of Public Health of Montenegro, Podgorica, Montenegro; 3 National Institute of Public Health of Slovenia, Ljubljana, Slovenia; 4 Institute of Public Health of Republika Srpska, Banja Luka, Bosnia and Herzegovina

## Abstract

**Background:**

Alcohol use is recognized as an important risk factor for more than 200 different diseases and injuries. In the Diagnostic statistic Manual- 5 (DSM-5) it is defined as ‘impaired control over alcohol consumption with chronic, heavy and often escalating pattern of alcohol use despite significant detrimental consequences to their overall health, the lives of their family members and friends and society in general’. The aim of this study was to describe the disability adjusted life years (DALY) associated with the alcohol use disorder in the Balkan countries in the period between 1990 and 2019.

**Methods:**

The study included the data on age-standardized DALY rate per 100,000 for alcohol use disorder in the period between 1990 and 2019 for ten Balkan countries (Albania, Bosnia and Herzegovina, Bulgaria, Croatia, Greece, Montenegro, North Macedonia, Romania, Serbia and Slovenia) from the Institute of Health Metrics and Evaluation. We acknowledge the support from the COST Action 18218 - European Burden of Disease Network.

**Results:**

The highest age-standardized DALY rate in 1990 was in Romania 484.03 per 100,000 (95% CI: 394.68-594.99), while the highest age-standardized DALY rate per 100,000 in 2019 was in Slovenia 427.75 (95% CI: 332.28-543.83). Along with Slovenia, only country that recorded the increase in the age-standardized DALY rates in the period between 1990 and 2019 was Albania, but the increase was only marginal (DALY per 100,000 increased from 174.24, 95% CI: 115.07-244.76 to 187.92, 95% CI: 127.26-262.41). The lowest burden measured in age-standardized DALY rates per 100,000 in 1990 was in Albania, and in 2019 was in Greece- 174.68, 95% CI: 113.66-251.34.

**Conclusions:**

In the majority of the Balkan countries the age-standardized DALY rates per 100,000 for alcohol use disorder decreased in the observed period. The increase is observed for Slovenia and Albania, with the more apparent increase in Slovenia.

**Key messages:**

- The age-standardized DALY rates for alcohol use disorder decreased in the majority of Balkan countries between 1990 and 2019.
- The age-standardized DALY rate increased in Slovenia and Albania between 1990 and 2019.

